# fNIRS Feasibility to Measure Brain Oxygenation Patterns of the Motor Cortex in Relation to Massage and Reflex Locomotion Therapy in Babies

**DOI:** 10.3390/jcm14113818

**Published:** 2025-05-29

**Authors:** Rocío Llamas-Ramos, Juan Luis Sánchez-González, Jorge Juan Alvarado-Omenat, Ismael Sanz-Esteban, J. Ignacio Serrano, Inés Llamas-Ramos

**Affiliations:** 1 Department of Nursing and Physiotherapy, Universidad de Salamanca, 37007 Salamanca, Spain; 2 Instituto de Investigación Biomédica de Salamanca (IBSAL), 37007 Salamanca, Spain; 3 FisioSport Salamanca, 37008 Salamanca, Spain; 4 Neurosciences and Physical Therapy Research Group, Department of Physiotherapy, Faculty of Sport Sciences, Universidad Europea de Madrid, 28670 Madrid, Spain; 5 Computational Modeling of Intelligence (COMODIN) Group, Center for Automation and Robotics, CSIC-UPM. Ctra. Campo Real km 0.200, Arganda del Rey, 28500 Madrid, Spain; 6 University Hospital of Salamanca, 37007 Salamanca, Spain

**Keywords:** babies, fNIRS, massage, reflex locomotion therapy, brain oxygenation

## Abstract

**Background:** Newborns’ plasticity allows the brain to adapt and reorganize in response to external stimuli; therefore, tactile stimuli could generate brain changes. The objective of this study was to verify the feasibility of using fNIRS to measure the degree of brain oxygenation with tactile techniques in babies. **Methods:** Oxygenation was recorded continuously and bilaterally before, during, and after the interventions (massage protocol and Reflex Locomotion Therapy) with functional near-infrared spectroscopy in 11-week-old babies. **Results:** Preliminary data suggested that the massage intervention decreased the activity bilaterally (first minute of the intervention) and then increased it bilaterally (second minute), where it continued to increase in the left hemisphere (third minute) before decreasing bilaterally (fourth minute). Finally, the activity continued to decrease in the right hemisphere but increased in the most dorsal area of the left hemisphere (fifth minute). For the Reflex Locomotion intervention, the activity substantially increased bilaterally (first minute of the intervention) and then decreased bilaterally, but more pronouncedly in the left hemisphere (second minute). Then, the activity decreased to pre-intervention values (third minute) and increased bilaterally again, but pronouncedly in the right hemisphere (fourth minute). In the fifth minute, the activity in the right hemisphere drastically decreased, but it increased in the left hemisphere. During the post-intervention resting period, in the massage intervention, the activity increased in the right hemisphere and in the most ventral part of the left hemisphere; in Reflex Locomotion Therapy, the activity decreased only in the left hemisphere. **Conclusions:** Both techniques achieve a potential increase in oxyhemoglobin concentration bilaterally during stimulation, but while the effects decrease with Reflex Locomotion Therapy, the effects are maintained with massage. More studies are needed to establish the neurophysiological basis of these therapies in pediatrics.

## 1. Introduction

Neuroplasticity is an ongoing short-, medium-, and long-term process in which the remodeling of neurosynaptic maps occurs, thus optimizing the functioning of brain networks during phylogeny, ontogeny, and beyond [1]. Plasticity in newborns refers to the ability of the developing brain to adapt and reorganize in response to external experiences and stimuli. During the first years of life, the brain shows remarkable flexibility that facilitates learning and adaptation, especially in areas such as language and sensory perception [2]. This period of high neuronal plasticity is essential for functional recovery in case of early injury, as it allows other brain areas to take over functions that might have been compromised [3]. However, this plasticity is also sensitive to environmental factors, implying that both enriching and adverse experiences can have long-lasting effects on infant brain development [4].

One of the main tools for measuring plasticity in newborns is near-infrared spectroscopy. Near-infrared spectroscopy (NIRS) is a non-invasive technique that has been fundamental in the study of brain plasticity in newborns. NIRS measures oxygenation in the brain, making it possible to analyze neuronal activity in real time and in a safe manner, which is especially important in such vulnerable populations as neonates. In recent studies, NIRS has demonstrated how exposure to specific sensory stimuli can modulate brain activity in areas such as the visual and auditory cortex, reflecting the remarkable plasticity of the neonatal brain [5].

Thanks to its ability to detect changes in brain hemodynamics, NIRS is useful for observing how newborn brains respond to linguistic and social stimuli, providing information about the development of functions such as facial recognition or voice perception, essential skills in social interaction [6]. These studies suggest that activity patterns measured by NIRS not only reflect present brain function but can also predict developmental and adaptive trajectories in response to early interventions or stimuli [7].

Newborn massage has been shown to have positive effects on brain development as measured by techniques such as functional near-infrared spectroscopy (fNIRS) [8]. Recent studies using fNIRS indicate that massage can increase oxygenation and blood flow in specific regions of the brain, especially in areas related to sensory perception and emotional regulation [9]. Other studies have shown that massage differentially activates sensorimotor areas and connections between the cortex and the limbic system, which could be related to the regulation of stress responses and the promotion of calmness and sleep [10]. This brain response observed with fNIRS underscores the importance of tactile experiences in neonates and suggests that interventions such as massage may play a crucial role in early nervous system development by modulating brain reactivity to external stimuli. Massage therapy exerts its effects through a combination of peripheral and central mechanisms. It has been shown to activate mechanoreceptors in the skin and muscles, resulting in increased parasympathetic activity and decreased sympathetic output, thereby promoting relaxation and reducing stress [11]. Additionally, massage reduces alpha motoneuron excitability, as evidenced by decreased H-reflex amplitude [12]. These physiological responses are associated with improved circulation, reduced muscle tension, and modulation of the hypothalamic–pituitary–adrenal axis [13].

Another therapy whose impact on neuronal plasticity measured with fNIRS has been studied is Reflex Locomotion Therapy. Sánchez-González et al. [14] demonstrated that through the therapy, there is an increase in brain activity in fundamental and essential areas for sensory processing, motor planning, and control. Reflex Locomotion Therapy is based on the activation of innate motor patterns through pressure stimulation of specific zones, which is believed to trigger central pattern generators (CPGs) in the brainstem and spinal cord. Clinical studies have suggested that this approach can induce neuroplastic changes and enhance motor coordination in individuals with neurological impairments and also supports the efficacy of Vojta therapy in activating reflex locomotion pathways [15]. Therefore, the following study is proposed to test the feasibility of using fNIRS to measure the degree of oxygenation with massage and Reflex Locomotion Therapy and the potential differences between these techniques so that they can be postulated as potential treatments in the development and brain maturation of infants and especially of the premature population.

## 2. Study Objectives

The study has the following objectives:

- To test the feasibility of using fNIRS in the measurement of brain oxygenation in healthy and preterm babies.
- To test the potential differences in brain oxygenation between massage therapy and Reflex Locomotion Therapy.
- To test potential sex differences in brain oxygenation after tactile stimuli.

## 3. Materials and Methods

### 3.1. Design

The study will be a randomized clinical trial, which will be randomly divided into two groups (massage and Reflex Locomotion Therapy).

### 3.2. Sample

Twenty babies born at term and without pathologies will be selected for the implementation of the study. The exclusion criterion will be any complication suffered during pregnancy and/or delivery.

### 3.3. Ethical Considerations

The parents of all participants will be informed of the objectives and development of the study and will sign an informed consent form to be included in the study. The study will follow the criteria established by the Helsinki Declaration and has obtained the approval of the ethics committee of the University of Salamanca (Code 806), and in accordance with the Data Protection Act, all data will be coded to guarantee the anonymity of the participants.

### 3.4. Material

**Figure 1:**
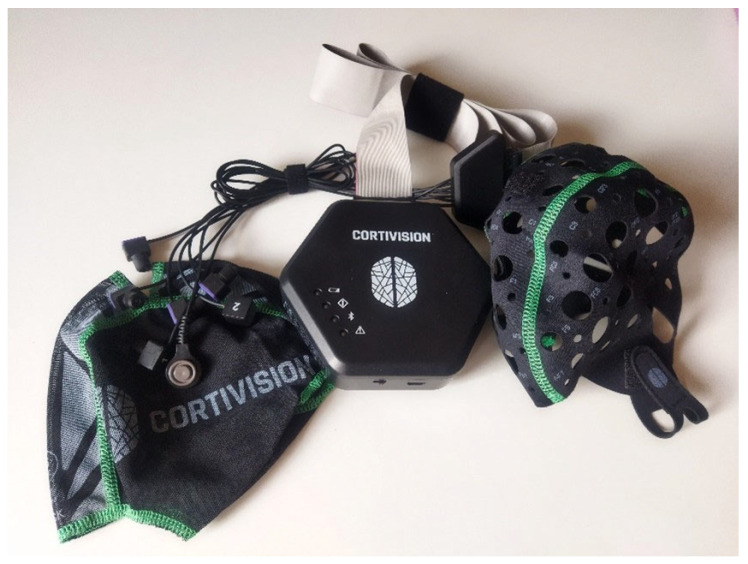
Cortivision photoncap type C20.

Cortivision photoncap type C20 (Baby kit) (Cortivision) has been selected to measure cerebral oxygen saturation (Figure 1).

**Figure 2:**
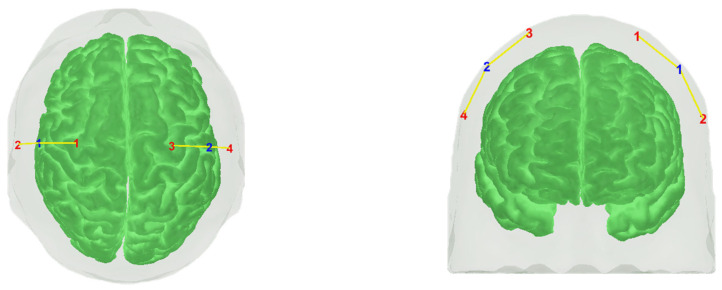
Schematic of the optode and its location versus the head model. Emitters: red numbers; detectors: blue numbers; left: top view; right: front view (Red numbers 1, 2, 3, and 4 are the optodes and blue numbers 1 and 2 de detectors, yellow lines mean the channels).

This measurement system continuously and non-invasively records HbO in the region covered by the sensor. The fNIRS helmet consists of a light-emitting diode system with four emitting optodes that generate two wavelengths in the near-infrared spectrum (730–810 nm), and one detector per hemisphere. On the right hemisphere, the configuration includes a detector at site C4 and emitters at sites C2 and C6. On the left hemisphere, the detector is positioned at C3, with emitters at C1 and C5. These photodetectors are located at a distance of 3 and 4 cm each from the emitting light source (Figure 2).

HbO will be recorded continuously and bilaterally (right and left somatosensory areas) before the stimulation, during the stimulation, and after the intervention.

### 3.5. Variables

The demographic variables related to the mother′s lifestyle are marital status, educational level, occupation, smoking, alcohol intake, physical activity and prescription of pharmacological treatment, age at menarche, and family history.

The primary outcome is the cerebral oxygenation values recorded by NIRS before, during, and after the application of massage and Reflex Locomotion Therapy.

NIRS measures changes in the concentration of oxyhemoglobin (HbO) and deoxyhemoglobin (HHb) molecules. By means of a single source emitting two wavelengths of near-infrared light and a single detector, it is possible to determine changes in the concentration of these molecules in the tissue through which the light has traveled. This capability will make it possible to study the functional response of the brain to external stimuli.

### 3.6. Procedure

Both interventions will be received by all participants at home to ensure quiet conditions, in a well-lit room with a pleasant temperature that allows the treatment techniques to be performed on the baby with the extremities and trunk uncovered.

One of the subjects will receive the massage protocol, applied by a physical therapist with extensive clinical experience in such therapy, while another researcher will be present throughout the procedure to control the monitor, in case any adverse events occur or the connection to the device is lost. The massage will begin by positioning the infant in the supine position, and the NIRS cap will be placed on the infant. According to the previously established protocol [16], 5 min of basal activity will be recorded with the baby in the arms before continuing with the application of the massage itself by performing passes with a total duration of 1 min in each body region starting with the right lower extremity, left lower extremity, right upper extremity, left upper extremity, and finally the thorax and abdomen. Finally, after 5 min of rest at the end of the massage, the basal variables will be recorded, again with the infant held in the arms.

The other subject will receive the application of Reflex Locomotion Therapy. The therapy will begin by positioning the baby in the supine position, and the NIRS cap will be placed on the baby. According to the previously established protocol [16], 5 min of basal activity will be recorded with the baby in the arms before continuing with the application of the intervention. Stimulation of the right pectoral point (between the 6th and 7th or 7th and 8th ribs) will be performed for 2 min; after 1 min of rest, the left pectoral point (between the 6th and 7th or 7th and 8th ribs) will be stimulated for 2 min. Finally, 5 more minutes rest will be recorded after the end of the technique with the infant held in the arms.

### 3.7. Data Processing

All data will be coded to ensure anonymity and analyzed by a blinded third researcher who will not be present at the assessments.

The fNIRS signal in *.snirf format will be pre-processed with Homer3 (Boston University; 2009) [17]. The pre-processing pipeline used will be the most robust and accurate one determined by Gemignani and Gervain [18] for fNIRS infant data. Specifically, pipeline A will be used, consisting of conversion to optical density units (hmrIntensity2OD function), determination of de/oxygenated hemoglobin concentration (hmrOD2Conc function), band-pass filtering (hmrBandpassFilt function: 0.01 Hz and 0.7 Hz, low-pass and high-pass cutoff, respectively), and correction of motion artifacts (hmrMotionCorrectPCSrecurse function: tMotion = 0.2, tMask = 1.0, StdevThresh = 50, and AmpThresh = 0.1). Finally, concentrations will be averaged for each time interval (hmrBlockAvg function).

Optode probes and HbO changes will be projected into a cortex template with AtlasViewer [19].

### 3.8. Statistical Analysis

For each optode, a mixed ANOVA will be used to test the effects of time interval, type of intervention, and their interaction. Greenhouse–Geisser correction will be applied in the case of a lack of sphericity as revealed by Mauchly’s test. Bonferroni correction will be applied to the statistical significance of the pairwise post hoc comparison between time intervals. Effect size will be reported as partial eta squared (η_p_^2^). The software IBM SPSS Statistics for Windows v29.0.2.0 (Armonk, NY, USA: IBM Corp) will be used for the mentioned analyses.

## 4. Results

Preliminary data collection was carried out to check the feasibility of the study. The pilot sample consisted of two female subjects with the same corrected gestational age of 11 weeks. Both did not present any pathologies or complications during pregnancy or delivery. Likewise, the mothers did not present any pathologies or prescribed pharmacological treatments. There were no complications arising from the performance of any of the interventions.

**Figure 3:**
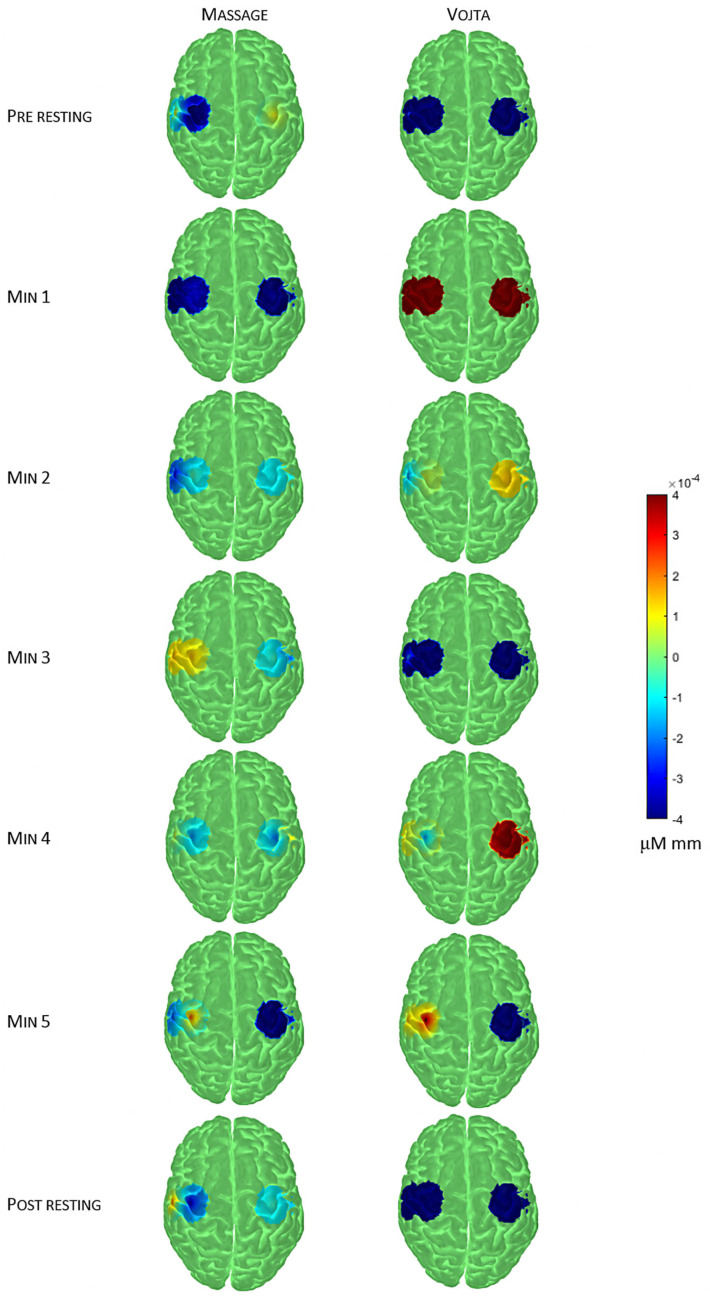
Average oxygenated hemoglobin concentration (HbO) for each time interval (row) and for each intervention (columns) projected over standard cortex template.

Figure 3 shows how the cortical activity over the motor cortex varies differently depending on the intervention. For the massage intervention, the activity decreased bilaterally during the first minute of the intervention. Then, it increased bilaterally during the second minute. In the third minute, the activity only continued to increase in the left hemisphere. Next, it decreased bilaterally during the fourth minute. In the fifth minute, the activity continued to decrease in the right hemisphere, but it increased in the most dorsal area of the left hemisphere. Finally, during the post-intervention resting period, the activity increased in the right hemisphere and in the most ventral part of the left hemisphere. For the Vojta intervention, during the first minute of the intervention, the activity substantially increased bilaterally. In the second minute, the activity decreased bilaterally, but more pronouncedly in the left hemisphere. Then, the activity decreased to pre-intervention values. In the fourth minute, the activity increased bilaterally again, but pronouncedly in the right hemisphere. In the fifth minute, the activity in the right hemisphere drastically decreased, but it increased in the left hemisphere. Finally, the activity decreased only in the left hemisphere during the post-intervention resting period.

Both subjects only showed similar patterns of HbO concentration during the fifth minute. The Vojta intervention reached a higher level of activation bilaterally during the intervention. The massage intervention, on the contrary, showed higher activation in the left hemisphere during the intervention, which was partly maintained during the post-intervention resting period in the most ventral part, but was always lower than in the Vojta intervention. The HbO patterns of both subjects during the pre-intervention resting period were different, with the massage subject presenting more activation in general and in the right hemisphere locally, contrary to the Vojta subject. Finally, the effect of the intervention with respect to the pre-intervention period was also different for each intervention. The massage subject showed a mild decrease in activation in the right hemisphere and a mild increase in the most ventral area of the left hemisphere. The Vojta subject went back to pre-intervention activation values bilaterally, or even lower.

With regard to the HbO concentration normalized with respect to the pre-intervention resting period, the left superior optode (1-1) presented statistically significant main effects of intervention, F(1,3640) = 14983.742, *p* < 0.001, η_p_^2^ = 0.805, and time interval, F(2.317,8432.455) = 4261.893, *p* < 0.001, η_p_^2^ = 0.539, as well as an interaction effect between both, F(2.317,8432.455) = 2199.685, *p* < 0.001, η_p_^2^ = 0.377. The Bonferroni-corrected post hoc analysis revealed that all pairwise differences between time intervals and interventions were statistically significant (*p* < 0.001, Table 1).

**Figure 4:**
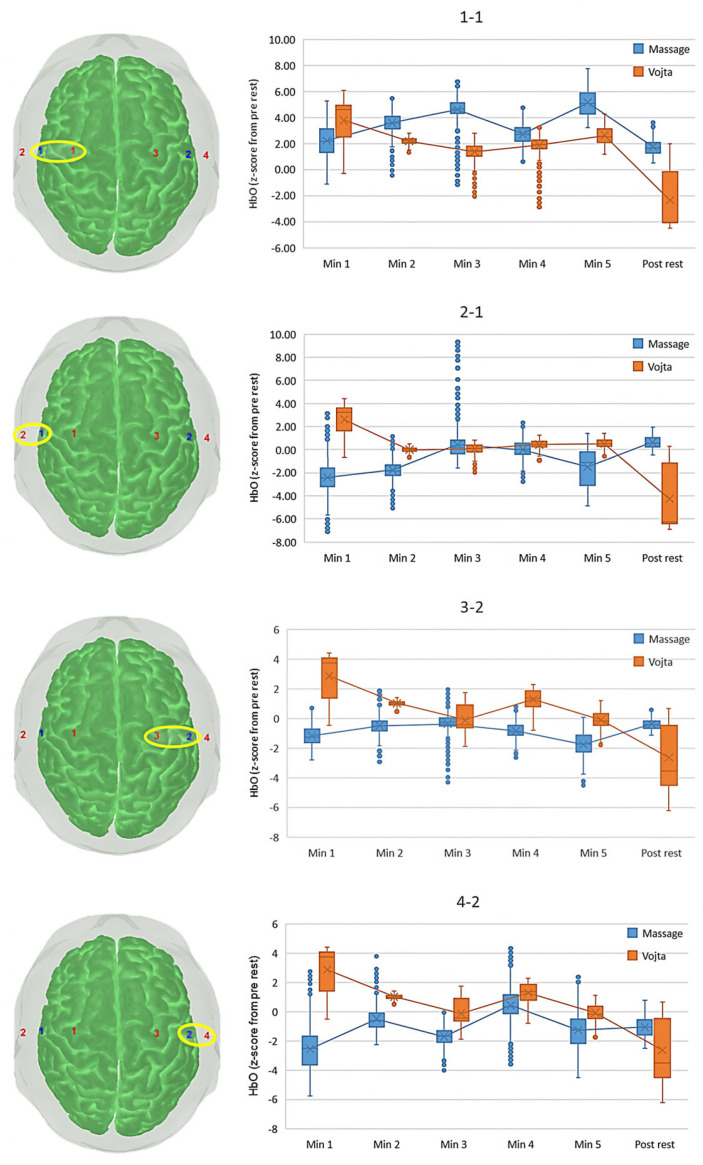
Box plots of the oxygenated hemoglobin concentration (HbO) normalized with respect to the pre-intervention resting period (z-scores) for each time interval (x-axis), each intervention (colors), and each pair of detector–emitter optodes (rows).

Figure 4 shows how the activation varies with respect to the pre-intervention resting period for each subject and each motor cortical area targeted. For the most rostral left area (1-1 optode), the massage showed an oscillating increase in activation during the intervention. The Vojta intervention, however, showed a u-shape activation with the lower value in the third minute (resting period) and a substantial decrease during the post-intervention period.

The most ventral left area (2-1 optode) presented statistically significant main effects of intervention, F(1,3640) = 4000.380, *p* < 0.001, η_p_^2^ = 0.542, and time interval, F(2.852,10379.896) = 964.317, *p* < 0.001, η_p_^2^ =.209, as well as an interaction effect between both, F(2.852,10379.896) = 4695.760, *p* < 0.001, η_p_^2^ = 0.563. The Bonferroni-corrected post hoc analysis showed that all pairwise differences between time intervals and interventions were statistically significant in the massage intervention and in the Vojta intervention except between minute 2 and minute 3 (Table 1). In this case, the interventions showed reversed patterns (Figure 4). The massage decreased the activity in the first minute, which then increased back to pre-intervention values to end with a slightly higher activation during the post-intervention period. The Vojta intervention, however, increased the activity in the first minute, which then decreased back to pre-intervention values until the post-intervention period, where the activity substantially decreased.

The right superior optode (3-2) presented statistically significant main effects of intervention, F(1,3640) = 29946.542, *p* < 0.001, η_p_^2^ = 0.892, and time interval, F(1.882,6850.328) = 2188.454, *p* < 0.001, η_p_^2^ = 0.375, as well as an interaction effect between both, F(1.882,6850.328) = 3252.696, *p* < 0.001, η_p_^2^ = 0.472. The Bonferroni-corrected post hoc analysis revealed that all pairwise differences between time intervals and interventions were statistically significant except between minute 3 and the post-rest interval during the massage intervention, and between minute 3 and minute 5 in the Vojta intervention (Table 1).

The right inferior optode (4-2) presented statistically significant main effects of intervention, F(1,3640) = 28089.548, *p* < 0.001, η_p_^2^ = 0.885, and time interval, F(2.558,9311.756) = 2476.019, *p* < 0.001, η_p_^2^ = 0.405, as well as an interaction effect between both, F(2.558,9311.756) = 2978.348, *p* < 0.001, η_p_^2^ = 0.450. The Bonferroni-corrected post hoc analysis showed that all pairwise differences between time intervals and interventions were statistically significant except between minute 3 and minute 5 in the Vojta intervention (Table 1).

In any case, both the rostral and ventral areas of the right hemisphere presented a similar tendency for each of the interventions (Figure 4). In the massage, the activation decreased in the first minute and kept oscillating but was always lower than in the pre-intervention period. In the Vojta intervention, the activation increased in the first minute and then monotonously decreased, ending with a lower activation than in the pre-intervention period.

## 5. Discussion

The purpose of this study was to test the feasibility of using fNIRS to measure the degree of oxygenation and the differences between each of the proposed techniques: massage and Reflex Locomotion Therapy. Our preliminary results show that fNIRS could demonstrate the potential existence of different brain oxygenation patterns in the pediatric population depending on the tactile stimulus or technique applied.

One of the first systems to develop in newborns is the sensory system, which is related to the perception of the individual’s own body as well as the surrounding environment [20]. Knowledge of these effects could lay the foundation for the development of tacit processing as well as opening the door to new early intervention [21]. Given the paucity of published information on brain responses to tactile stimuli, Shibata et al. [20] conducted a study in which they evaluated the brain response to different sensory stimuli in newborn infants up to 9 days old using NIRS technology by collecting signals from the parietal, temporal, and occipital areas to gather information on tactile, auditory, and visual stimuli. The results showed that these stimuli activated the corresponding primary areas, with tactile stimuli activating the largest areas, thus demonstrating different reactions to different stimuli, as well as the importance of tactile stimuli in early developmental stages. Other authors have shown how certain brain areas, specifically the superior temporal area, are activated, in this case with affective touch, which is responsible for the processing of socioemotional stimuli not only in infants but also in children and adults [22], demonstrating that brain activation at the temporal level by these stimuli may be related to individual differences in the affective reaction to touch. Similarly, Jonsson et al. [23] planned a study with affective and non-affective touch in infants as young as 2 months of age, showing activation of the temporal region, while non-affective touch—fast touching—reflected changes in the insula. Motivated by these results, in the present study, we selected two different types of tactile stimuli that have shown positive effects to postulate them as the best alternatives for stimulation and even treatment in the case of pathologies.

Regarding the selected stimulus, Fuchino et al. [24] selected a tactile vibration stimulus with prolonged application on the extremities to test the changes in HbO, while other authors [22] applied the stimuli on the upper extremities, specifically on the forearm, while watching a silent movie. The first study was conducted with term babies and infants up to 10 months of age, while the second involved infants who were 12 months of age. Despite being a different stimulus, the application of the present stimulus was also performed on the extremities in relation to the massage technique, while the Vojta therapy was applied on the thorax.

Hemodynamic changes related to neuronal activity have been used as an alternative to measure brain function in the early stages of development. Whether these hemodynamic changes coincide with those observed in the adult population has also been analyzed, demonstrating that they are different [24]. In the present study, only infants of almost 3 months of age were evaluated, so we cannot confirm these results.

The most used parameters have been changes in HbO and HHb [22,24]. Miguel et al. [22] observed a significant increase in the hemodynamic response of HbO to affective touch in the temporal region of infants with less aversive behavioral responses to tactile stimuli. On the other hand, Fuchino et al. [24] demonstrated that age modified the HbO amplitude in the sensorimotor motor area but not in the supplementary motor area; the amplitude showed a maximum decrease in the 1–2-month age group and subsequently increased with development. This could reflect developmental changes in neural activity, vascularization, and oxygenation. In the present study, the sample was of the same age, so these differences could not be observed.

Vojta therapy has been shown to activate reflex movement patterns, as well as brain neural networks. However, the studies that have evaluated this technique with fNIRS have been carried out in adults [14]. The results have shown an increase in concentration from the basal phase to the first resting period in the right hemisphere, contralateral to the stimulation area. Despite being very different samples, the existence of changes in the brain after tactile stimulation with an increase in the activation of sensory areas has been demonstrated in the same way. In relation to our sample, the greatest activation occurred bilaterally in the stimulation phase, but it decreased at the end of the stimulation. This suggests a transient effect of Reflex Locomotion Therapy or the Vojta method on cortical hemodynamics. On the contrary, in the massage intervention, this increase in activation was achieved during the stimulation but was also maintained after it, suggesting more prolonged cortical engagement or a delayed neuromodulatory response. This technique has already been evaluated in adults with fNIRS technology to determine its effects at the cerebral level [9]. Other authors such as Lai et al. [25] proposed a study where they evaluated these effects in preterm infants; however, the results have not been found. Finally, massage has also been analyzed in both newborn babies and their mothers, concluding that it both favors cerebral oxygenation and strengthens the mother–infant bond [26].

For other modalities of tactile stimulation such as foot massage [27] or even Chinese massage [28], unfortunately all the investigations have been carried out in adults or in populations with pathologies such as cancer [29]; in addition, this last publication used magnetic resonance technology, which prevents us from comparing the results.

Among the limitations of the present study is the small sample. However, given the nature of the selected sample, it was decided to obtain preliminary results that justify the implementation of a study with a larger sample without causing harm to the participants. In this line, several values such as blood pressure, heart rate, and breath rate have not been considered, but they could be taken into account in future studies. Another limitation is the absence of short separation channels, which can considerably reduce the confounding factor of the hemodynamic changes in the scalp vasculature. Nonetheless, the averaged explained variance of the scalp hemodynamics on the normalized HbO signal has been proven to be in the range of 7–10% [30]. Given that the significant differences found in the present study are far higher than that range, the results are not substantially affected. Moreover, in contrast to other fNIRS-based studies, this study focused solely on oxygenated hemoglobin (HbO) measurements rather than deoxygenated hemoglobin (HHb). However, HbO provides several advantages when studying infants. HbO signals typically have a 2–3 times higher amplitude than HHb signals in infants [5,31]. This creates a better signal-to-noise ratio, which is particularly important when working with challenging infant populations where data quality is often compromised by movement artifacts, as in the present study. Moreover, in developing infant brains, the neurovascular coupling mechanisms are still maturing [32]. Research suggests that HbO responses are more consistent and reliable indicators of neural activity in this population, as the metabolic dynamics of HHb may be less tightly coupled to neural events in immature brains [33]. In addition, HbO measures have demonstrated particular sensitivity to developmental changes in cognitive function and brain maturation in longitudinal infant studies [34,35]. Finally, there are substantial precedents in the developmental cognitive neuroscience literature for primarily reporting HbO, creating better comparability across studies [36,37].

## 6. Future Lines of Research

In future lines of research, a randomized clinicial trial will be carried out, where the variety and age of the subjects as well as the sample size will be expanded to obtain reliable results that postulate these stimulation techniques as valid or even as potential treatments in pediatric populations with developmental limitations or delays. Also, short separation channels will be included to reduce the influence of systemic hemodynamics.

## 7. Conclusions

fNIRS has proven to be a valid and reliable method to measure oxyhemoglobin and deoxyhemoglobin concentrations derived from the application of massage and Reflex Locomotion Therapy. Both techniques could achieve an increase in oxyhemoglobin concentration bilaterally during stimulation, but while the effects decrease with Reflex Locomotion Therapy after the intervention, the effects are maintained with massage. More studies are needed to establish the neurophysiological basis of these two therapeutic modalities in the pediatric population.

## Figures and Tables

**Table 1:** Average (standard deviation) values of the oxygenated hemoglobin changes (HbO) normalized with respect to the pre-intervention resting periods (z-scores) for each optode and time interval in the rows and each intervention in the columns. Bonferroni-corrected post hoc *p*-values between all pairs of time intervals and interventions are reported for each optode.

Optode	Time Interval	Massage	Vojta	*p*-Values (Post Hoc)
Left superior (1-1)	Min 1	2.156 (1.479)	3.816 (1.804)	<0.001
	Min 2	3.577 (0.814)	2.199 (0.251)	<0.001
	Min 3	4.618 (1.032)	1.317 (0.639)	<0.001
	Min 4	2.687 (0.841)	1.838 (0.660)	<0.001
	Min 5	5.112 (1.006)	2.726 (0.598)	<0.001
	Post rest	1.752 (0.693)	−1.709 (2.546)	<0.001
	*p*-value (post hoc)	<0.001	<0.001	
Left inferior (2-1)	Min 1	−2.369 (1.596)	2.688 (1.341)	<0.001
	Min 2	−1.782 (0.843)	−0.011 (0.197) ^§^	<0.001
	Min 3	0.438 (1.436)	0.017 (0.425) ^§^	<0.001
	Min 4	−0.069 (1.013)	0.422 (0.408)	<0.001
	Min 5	−1.453 (1.645)	0.584 (0.379)	<0.001
	Post rest	0.645 (0.505)	−3.603 (2.923)	<0.001
	*p*-value (post hoc)	<0.001; 3-post rest *p* = 0.002	<0.001	
Right superior (3-2)	Min 1	−1.173 (0.706)	2.909 (1.581)	<0.001
	Min 2	−0.520 (0.527)	1.012 (0.147)	<0.001
	Min 3	−0.397 (0.766) ^§^	−0.155 (0.843) ^§^	<0.001
	Min 4	−0.851 (0.490)	1.332 (0.638)	<0.001
	Min 5	−1.771 (0.728)	−1.104 (0.547) ^§^	<0.001
	Post rest	−0.389 (0.357) ^§^	−2.140 (2.315)	<0.001
	*p*-value (post hoc)	<0.001; 2-post rest *p* = 0.018	<0.001 but Min3-Min5	
Right inferior (4-2)	Min 1	−2.515 (1.521)	2.912 (1.578)	*p* < 0.001
	Min 2	−0.511 (0.782)	1.019 (0.145)	*p* < 0.001
	Min 3	−1.834 (0.690)	−0.164 (0.840) ^§^	*p* < 0.001
	Min 4	0.790 (1.100)	1.328 (0.636)	*p* < 0.001
	Min 5	−1.616 (1.021)	−0.095 (0.545) ^§^	*p* < 0.001
	Post rest	−1.012 (0.606)	−2.144 (2.311)	*p* < 0.001
	*p*-value (post hoc)	<0.001	<0.001	

^§^ Pair differences not statistically significant for the corresponding optode.

## Data Availability

The data sets generated and/or analyzed during the current study are available from the corresponding author on reasonable request.

## Abbreviations

The following abbreviations are used in this manuscript:

HHb	Deoxyhemoglobin
fNIRS	Functional near-infrared spectroscopy
HbO	Oxygenated hemoglobin
NIRS	Near-infrared spectroscopy
rSO_2_	Regional oxygen saturation
